# Meteorin-like levels of oral fluids in periodontal health and diseases: a comparative cross-sectional study

**DOI:** 10.1007/s00784-025-06222-7

**Published:** 2025-02-18

**Authors:** Ebru Saglam, Ayse Toraman, Levent Savran, Mehmet Saglam, Serhat Koseoglu

**Affiliations:** 1 Department of Periodontology, Faculty of Dentistry, Istanbul Medeniyet University, Istanbul, 34956 Turkey; 2 Department of Periodontology, Faculty of Hamidiye Dentistry, Saglik Bilimleri University, Istanbul, 34668 Turkey; 3 Department of Periodontology, Faculty of Dentistry, Izmir Katip Celebi University, Izmir, 35620 Turkey

**Keywords:** Cytokine, Gingival crevicular fluid, Interleukins, Periodontitis, Saliva

## Abstract

**Objectives:**

Cytokines are critical role in the pathogenesis of periodontal diseases. Meteorin-like (Metrnl), a protein released in the oral mucosa, is a novel cytokine associated with various inflammatory diseases. This study aimed to evaluate levels of salivary and gingival crevicular fluid (GCF), interleukin − 1 beta (IL-1β), interleukin − 10 (IL-10), and Metrnl in periodontal disease.

**Materials and methods:**

It was formed three groups of 20 individuals each: the healthy, gingivitis, and periodontitis (Stage III, Grades A and B) groups. The enzyme-linked immunosorbent assay method measured IL-1β, IL-10, and Metrnl levels in salivary and GCF samples. Clinical periodontal measurement including probing pocket depth (PD), clinical attachment loss (CAL), plaque index (PI), gingival index (GI) and percentage of bleeding on probing (%BOP); and biochemical parameters were evaluated using statistical analysis.

**Results:**

Salivary concentrations and GCF total amount of IL-1β were significantly higher in the periodontal disease groups (*p* < 0.001). There was no significant difference among the groups in either salivary concentrations or GCF total amount of IL-10 (*p* > 0.05). Salivary Metrnl concentrations were significantly lower in the periodontitis group (*p* = 0.002). Salivary Metrnl concentrations had significant negative correlations with %BOP, PD, and CAL (*p* < 0.05). GCF total amount of Metrnl had significant negative correlations with GCF total amount of IL-1β (*p* = 0.03). The receiver operating characteristics curve showed: area under the curve (AUC) = 0.731 for salivary Metrnl concentrations to discriminate periodontitis from periodontal health and gingivitis.

**Conclusion:**

Based on the findings of this study, Metrnl, as an immunoregulatory cytokine, may have an important relationship with periodontitis. Additional studies are needed to fully elucidate the functional effect of Metrnl in periodontal disease.

**Clinical relevance:**

**Background** In knock-out experimental studies, it has been reported that Metrnl acts as an inflammation-related immunoregulatory cytokine. The findings relating it to inflammatory diseases support the idea that Metrnl may play a role in the pathogenesis of a variety of inflammatory diseases.

**Added value of this study:**

Although Metrnl is a protein abundantly secreted in the oral mucosa, to the authors’ knowledge there has been no study evaluating it in gingivitis and periodontitis, inflammatory diseases. In this study, the levels of Metrnl in oral fluids, such as GCF and saliva, were examined in the presence of different periodontal diseases.

**Clinical implications:**

Metrnl can be useful in distinguishing periodontal destruction from periodontal health.

## Introduction

Periodontal diseases are destructive inflammatory disorders affecting dental support tissues. At the root of this is dysbiosis, which develops as a result of interactions among polymicrobial communities, host, genetics, and acquired risk factors [1]. Polymicrobial communities and dysbiosis adversely affect the host’s immuno-inflammatory system [2]. Destruction of periodontal tissues occurs because the balance between the pro- and anti-inflammatory mediators deteriorates [3, 4].

Mediators such as cytokines, chemokines, and metalloproteinases are significantly increased in periodontal tissues and gingival crevicular fluid (GCF) in periodontitis [3]. Cytokines are low molecular weight proteins that play important roles in the initiation and subsequent stages of inflammation, regulating the inflammatory response’s amplitude and duration [5]. A cytokine of the first innate response to occur in the pathogenesis pathway of periodontal disease is interleukin-1 beta (IL-1β) [5, 6] an important pro-inflammatory mediator characteristically associated with inflammatory cell migration and osteoclastogenesis [5, 7]. Increased levels of IL-1β, a precursor of other cytokines in the inflammatory process, play an important role in the onset and development of periodontitis pathogenesis. It also triggers several inflammatory reactions and promotes alveolar bone resorption [8]. Salivary concentrations and GCF total amount of IL-1β have been indicated to be higher in periodontitis patients than in periodontally healthy individuals [9–13].

Interleukin − 10 (IL-10), an anti-inflammatory cytokine, suppresses the immune response and supports the resolution of inflammation by inhibiting macrophages from presenting antigens and producing pro-inflammatory cytokines [14]. It also exerts antifibrotic activity by preventing angiogenesis, scar formation, and re-epithelialization and contributes to the regulation of wound healing [4]. IL-10 is thought to play a role in periodontitis by reducing the production of proinflammatory cytokines and stimulating the production of protective antibodies [15]. Previous studies have reported different results regarding salivary concentrations and GCF total amount of IL-10, such as increased [11, 16, 17], no differ [18, 19], and decreased [20, 21] in periodontal diseases.

Meteorin-like (Metrnl) is a small protein (~ 27 KDa) involved in innate and adaptive immune-related functions and inflammatory responses [22], also called Meteorin-β, IL-41, Cometin, or Subfatin [23]. Metrnl can be selectively expressed by different cell types [24]. It is abundantly expressed in M2-polarized macrophages and barrier tissues (skin and mucous membranes) [25]. The oral mucosa, pharyngeal mucosa, and esophagus are among the top ten sites of Metrnl synthesis in the human body [26]. It reportedly assists in functions such as wound healing, tissue remodeling or Th2 responses, and participates in certain inflammatory responses [26, 27]. Metrnl exerts pleiotropic effects on inflammation, immunology, and metabolism [24]. Metrnl reduces inflammation in adipose tissue via peroxisome proliferator-activated receptor-Ƴ (PPARƳ), in skeletal muscles via AMP-activated protein kinase (AMPK) or peroxisome proliferator-activated receptor δ (PPARδ), and blocks nuclear factor kappa β (NF-kβ) [28].

In a study in knock-out mice, Metrnl was shown to be an immunoregulatory cytokine associated with inflammation [22]. Considering the studies that found a relationship between various systemic diseases such as psoriasis, rheumatoid arthritis, osteoarthritis, and cardiac dysfunction and Metrnl, there has no consensus yet on whether Mertnl levels are increasing or decreasing in biological fluids or tissues [22, 26, 29–37]. Besides this, there are increasing studies suggesting that Metrnl may play a role in the pathogenesis of inflammatory diseases [22, 26, 37, 38].

To our knowledge, there has no study to evaluate salivary concentrations or GCF total amount of Metrnl in periodontitis, an inflammatory disease. This study aimed to comparatively evaluate the levels of Metrnl, pro-inflammatory (IL-1β), and anti-inflammatory (IL-10) cytokines in salivary and GCF samples in the presence of periodontal disease.

## Methods

The Izmir Katip Çelebi University Clinical Research Ethics Committee granted approval (42/2019). After all participants were informed about the study’s aim and method, they signed informed consent forms. All clinical measurements were performed in accordance with the Declaration of Helsinki. The study was registered on ClinicalTrials.gov (No: NCT04402996).

### Patient selection

Between September 2020 and March 2021, anamnesis for eligibility for inclusion in the study was taken from individuals who were clinically and radiologically diagnosed with periodontitis at the Faculty of Dentistry, Izmir Katip Celebi University.

Systemically healthy patients with at least two permanent teeth in each half jaw, who had never smoked, and were not pregnant, lactating, menstruating, or menopausal were included in the study. Patients who had used continuous medication, antibiotics, and anti-inflammatory or systemic corticosteroids in the last six months were excluded. Individuals with a history of periodontal treatment in the last six months, requiring restorative and/or endodontic treatment, who had orthodontic appliances, removable dentures, or crown-bridge restoration in the tooth from which GCF would be taken, or in the neighboring teeth, were excluded. Third molars were excluded from the evaluation.

Of the 97 individuals examined initially for this cross-sectional comparative study, 65 were eligible and met the inclusion criteria; five participants declined to take part. Thus, the total study population comprised 60 individuals. Participant were included to groups according to the order in which they applied to the clinic. No participants dropped out of the study.

Post hoc power analysis was performed with G*Power software (G*Power 3.1.9.7, Heinrich Heine-University, Dusseldorf, Germany) based on salivary Metrnl averages, which is the primary outcome of the study. Effect size was found as 0.512 (standard deviation: 20.93), α as 0.05, and power as 0.91.

### Dental examination

All clinical periodontal parameters were measured using a calibrated investigator (LS) with a periodontal probe (Williams, Hu-Friedy, Chicago, IL, USA). The intra-examiner exercise was performed for clinical attachment loss (CAL), Kappa value was 0.89.

Probing pocket depth (PD), CAL, plaque index (PI) [39], gingival index (GI) [40], and percentage of bleeding on probing (%BOP) [41] were measured on six surfaces of each tooth. PD is the distance from the gingival edge to the base of the gingival crevice/periodontal pocket while CAL is the distance from the enamel–cementum junction to the base of the periodontal pocket. Alveolar bone loss was evaluated on orthopantomography by the ratio of the distance between the enamel–cementum junction and the bone crest to the root length in mesial and distal regions.

Participants were divided into three groups according to the diagnostic criteria of Classification of Periodontal and Peri-implant Disease and Conditions [42, 43]. (1) Healthy (control) group (n:20): this group had no clinical signs of inflammation in periodontal tissues, an intact periodontium without interproximal CAL or radiographic bone loss, a PD of ≤ 3 mm, the number of BOP sites being less than 10%, and no history of periodontitis. (2) Gingivitis group (n:20): clinical signs of inflammation with a PD of ≤ 3 mm and ≥ 30%, the number of BOP sites included without CAL or radiographic bone destruction in periodontal tissues [42]. Periodontitis group (n:20): ≥ 30% teeth involved, PD ≥ 6 mm and CAL ≥ 5 mm, no more than four teeth lost due to periodontitis and radiographic bone loss > 33%. Grade was determined by the ratio of the percentage of radiographic bone loss of the tooth with the most severe bone loss radiographically to age (Grade A < 0.25, Grade B 0.25-1) [43]. The study included only individuals with generalized Stage III Grade A and B periodontitis.

### Salivary and GCF sampling

Sampling was performed one day after periodontal clinical measurements had been taken for standardization, after an overnight fast. Patients were asked to avoid brushing, eating, drinking, and chewing gum for at least two hours before sampling.

To take unstimulated whole saliva samples, patients were seated upright, head tilted forward, and saliva was deposited on the floor of the mouth for five minutes. This ensured that the accumulated saliva flowed passively through a sterile polypropylene tube [44]. It was transferred to a 50 mL centrifuge tube with the help of a sterile syringe and was centrifuged (Vacuette, Greiner Bio-One, Kremsmünster, Austria) at 800×g at 4 °C for 10 min to separate the debris. Supernatant was transferred to 1.5 mL Eppendorf tubes, sealed with paraffin tape and stored at -80 °C until the analysis [45].

To avoid contamination, the maxilla was chosen for sampling whenever possible. GCF samples were made using paper strips (Periopaper^®^, ProFlow, Inc., Amityville, NY, USA). Samples were obtained from two teeth, one with a single root and one with multiple roots. Samples were taken from the two regions with GI ≤ 1, PD ≤ 3, and BOP negative in the healthy group, from the two regions that were GI ≥ 2, PD ≤ 3, and BOP positive in the gingivitis group, and from two regions with GI ≥ 2 and positive BOP 5 mm or over with the deepest PD in the periodontitis group [45]. Before sampling, the relevant area was isolated with cotton rolls to prevent saliva contamination. The supragingival plaque was gently removed without touching the gingiva and the sampling areas were dried with compressed air. Paper strips contaminated with blood or saliva were not included. The strips were placed in the gingival pocket so that a slight resistance was felt, taking care not to cause mechanical trauma, and left for 30 s [45]. The GCF volume of the strips was measured with a calibrated electronic device (Periotron 8000, Harco Electronics, Winnipeg, Canada). Two strips obtained from two non-adjacent areas of each participant were placed in a propylene tube and pooled. Samples were stored at -80ºC until the analysis. Readings were converted to actual volume (microliters) with reference to the standard curve.

### Biochemical analyses

Salivary and GCF IL-1β, IL-10, and Metrnl measurements were carried out using the ELISA method with commercial kits (MyBioSource Human ELISA Kit, San Diego, California, USA for Il-1β; MyBioSource Human ELISA Kit, San Diego, California, USA for IL-10; R&D Systems, Human Meteorin-like/METRNL& Ancillary Reagent DuoSet ELISA, Minneapolis, USA for Metrnl.)

The ELISA kits protocol for IL-1β and IL-10; 100 µL of sample were added to each well and incubated at 37℃ for 90 min. The liquid were removed, 100 µL of Biotinylated Detection Ab were added and incubated at 37℃ for 1 h. Aspirated and washed 3 times. Added 100 µL of HRP Conjugate and incubated at 37℃ for 30 min. Aspirated and washed 5 times. 90 µL of Substrate Reagent were added, and incubated at 37℃ for 15 min.

The ELISA kits protocol for Metrnl; 100 µL of samples were added in Reagent Diluent, or an appropriate diluent, per well and incubated for 2 h at room temperature. The aspiration/wash was repeated. 100 µL of the Detection Antibody was added, diluted in Reagent Diluent, to each well, and incubated for 2 h at room temperature. The aspiration/wash was repeated. 100 µL of the working dilution of Streptavidin-HRP were added to each well and incubated for 20 min at room temperature. The aspiration/wash was repeated. 100 µL of Substrate Solution were added to each well, and incubated for 20 min at room temperature (avoid placing the plate in direct light).

After adding stop solution, the optical density was determined of each well immediately by a microplate reader set to 450 nm.

### Statistical analyses

Statistical analyses were performed using SPSS software version 22. Descriptive statistics were used in the presentation and evaluation of clinical, laboratory, and biochemical data. Kurtosis skewness values were used for normality assumption [46] and variance homogeneity was evaluated by Levene’s test. One-way ANOVA and Tukey tests were applied for parametric data, and Kruskal–Wallis and Tamhane tests were employed for non-parametric data to evaluate the differences among the groups. ROC curve analysis was used to estimate the diagnostic performance for IL-1β and Metrnl concentrations. The statistical significance of the results was evaluated at *p* < 0.05. Cytokine levels were presented as total amount for GCF (pg/30 s) and as concentrations for saliva (pg/mL).

## Results

### Clinical and demographic data

There were 60 participiant, 26 females and 34 males, aged 19–60 years were included (Table 1). The periodontitis group consisted of 10 grade A and 10 grade B participants. All groups were significantly different in terms of GI, %BOP, and PD, and the values increased from the healthy group to the periodontitis group (*p* < 0.001). Significantly higher PI values were observed in the gingivitis and periodontitis groups, with no significant difference between these two groups.

**Table 1:** Demographic and clinical parameters

	Healthy (*n*:20) Mean ± SD Median (min-max)	Gingivitis (n:20*)* Mean ± SD Median(min-max)	Periodontitis (*n*:20) Mean ± SD Median (min-max)	*p* value
Age (years)	26.27 ± 4.32 26.5 (20–32)	34.17 ± 10.9^a^ 36 (19–57)	42.33 ± 10.19^a, b^ 41.5 (22–60)	< 0.001
GI	0.39 ± 0.22 0.38 (0.08–0.83)	1.14 ± 0.20^a^ 1.45 (1.13–1.95)	1.7 ± 0.38^a, b^ 1.65 (1.11–2.61)	
PI	0.68 ± 0.3 0.7 (0.21–1.31)	1.39 ± 0.21^a^ 1.41 (0.96–1.76)	1.55 ± 0.3^a^ 1.58 (1.16–2.17)	
%BOP	5.51 ± 2.98 5.87 (0–9)	44.11 ± 10.82^a^ 39 (32–63)	59.34 ± 21.76^a, b^ 60.19 (32.69–100)	
PD (mm)	1.31 ± 0.22 1.39 (0.86–1.62)	2.02 ± 0.36^a^ 2.02 (1.30–2.81)	3.29 ± 0.64^a, b^ 3.28 (2.39–4.91)	
CAL (mm)	NS	NS	3.05 ± 0.62^a, b^ 3.07 (1.93–4.44)	
GCF volume (µL)	0.49 ± 0.24 0.5(0.11–0.87)	0.66 ± 0.22 0.64(0.32–1.19)	1.54 ± 0.17^a, b^ 1.59(1.19–1.78)	
Missing teeth	NS	0.18 ± 0.72	1.89 ± 1.74 ^a, b^	< 0.05

SD: Standart deviation

*p* < 0.001, *p* < 0.05 one way ANOVA test

a: statistically significant difference compared to the healthy group

b: statistically significant difference compared to the gingivitis group

GI, gingival index; PI, plaque index; BOP, bleeding on probing; PD, probing pocket depth; CAL, clinical attachment loss; GCF, gingival crevicular fluid

GCF volume values were significantly higher in the periodontitis group (*p* < 0.001).

### Biochemical data

#### Laboratory findings

Laboratory findings are summarized in Table 2.

**Table 2:** Salivary and GCF IL-1β, IL-10 and Metrnl Levels

	Healthy (*n*:20) Mean ± SD Median (min-max)	Gingivitis (*n*:20) Mean ± SD Median (min-max)	Periodontitis (*n*:20) Mean ± SD Median (min-max)	*P* value
Salivary IL-1β (pg/mL)	14.62 ± 2.44 14.29(10.64–19.61)	93.68 ± 34.52^a^ 92.66(24.64-151.13)	83.15 ± 84.47^a^ 59.51(10.06-315.61)	< 0.001
GCF total amount of IL-1β (pg/30 s)	5.70 ± 2.73 4.45(4.08–13.02)	9.79 ± 5.82^a^ 8.8(4.49–28.11)	16.29 ± 7.47^a, b^ 14.3(5.35–31.84)	< 0.001
GCF IL-1β concentration (pg/µL)	18.34 ± 19.98 9.7(4.82–83.19)	16.10 ± 11.64 13.3(9.12–53.91)	10.75 ± 5.3 9.48(3.36–23.59)	0.24
Salivary IL-10 (pg/mL)	14.08 ± 2.84 12.84(11.40–19.90)	14.78 ± 3.31 13.11(10.41–20.62)	18.27 ± 11.09 13.9(10.41–47.67)	0.78
GCF total amount of IL-10 (pg/30 s)	12.32 ± 16.63 6.16(5.55–57.77)	6.92 ± 1.8 6.6(5.55–13.49)	6.69 ± 0.68 6.5(5.82–7.9)	0.20
GCF IL-10 concentration (pg/µL)	30.56 ± 35.29 15.13(6.42-135.06)	11.57 ± 4.45^a^ 10.08(6.1-21.09)	4.37 ± 0.55^a^ 4.34(3.36–5.4)	0.001
Salivary Metrnl (pg/mL)	99.18 ± 10.8 100.91(82.34-123.97)	103.07 ± 20.06 107.5(62.43-135.21)	78.65 ± 27.8^a, b^ 82.2(28.49–117.5)	0.002
GCF total amount of Metrnl (pg/30 s)	80.18 ± 12.25 81.01(53.91-100.27)	75.2 ± 12.09 79.66(51.80-92.91)	73.61 ± 12.18 77.37(46.26–88.27)	0.25
GCF Metrnl concentration (pg/µL)	239.76 ± 203.73 167.15(82.79-817.05)	128.78 ± 53.67^a^ 128.48(67.6-254.72)	48.43 ± 10.35^a^ 107.15(25.99–67.54)	< 0.001

SD, standart deviation; GCF, gingival crevicular fluid; IL, interleukin

a: statistically significant difference compared to the healthy group, *p* < 0.05

b: statistically significant difference compared to the gingivitis group, *p* < 0.05

*p value*: For GCF total amount of IL-1β, salivary and GCF total amount of IL-10, Kruskal Wallis test was used while one-way ANOVA test used for other analysis

Salivary IL-1β concentrations were significantly higher in both the gingivitis and periodontitis groups when compared to healthy group (*p* < 0.001) (Table 2). However, there was no significant difference between these groups in terms of salivary IL-1β concentrations. The GCF total amount of IL-1β was significantly higher in both these groups than the healthy group. The GCF total amount of IL-1β was significantly higher in the periodontitis group compared to the gingivitis group (*p* < 0.001). There was no significant difference in GCF IL-1 β concentrations among the groups.

There was no significant difference among the groups for salivary IL-10 concentrations (*p* > 0.05). The GCF total amount of IL-10 was lower in the gingivitis and periodontitis groups, but no significant difference was observed among the groups (*p* > 0.05). The GCF IL-10 concentrations were significantly lower in the gingivitis and periodontitis groups than in the healthy group (*p* = 0.001) (Table 2).

Salivary Metrnl concentrations were significantly lower in the periodontitis group (*p* = 0.002). There was no significant difference between the gingivitis and healthy groups. Although the GCF total amount of Metrnl were higher in the healthy group, the differences among the groups were not significant. The GCF Metrnl concentrations were significantly lower in the gingivitis and periodontitis groups than in the healthy group (*p* < 0.001) (Table 2).

#### Correlations

Salivary IL-1β concentrations were positively correlated with all clinical periodontal parameters except CAL and the GCF total amount of IL-1β (*p* < 0.001). The GCF total amount of IL-1β was positively correlated with all clinical periodontal parameters and GCF total amount of IL-10 (*p* < 0.001 and *p* < 0.05, respectively). There was a positive correlation between GCF total amount of IL-10 and GI (*p* < 0.05) (Table 3).

**Table 3:** Correlations among biochemical parameters and periodontal clinical parameters

	GI		PI		%BOP		PD		CAL		GCF total amount of Metrnl (pg/30 s)		Salivary Metrnl (pg/ml)		GCF total amount of IL-10 (pg/30 s)		Salivary IL-10 (pg/ml)		GCF total amount of IL-1β (pg/30 s)	
	*r*	*p*	*r*	*p*	*r*	*p*	*r*	*p*	*r*	*p*	*r*	*p*	*r*	*p*	*r*	*p*	*r*	*p*	*r*	*p*
Salivary IL-1β (pg/ml)	0.641	< 0.001**	0.594	< 0.001**	0.550	< 0.001**	0.505	< 0.001**	0.234	0.091	-0.192	0.16	0.260	0.06	0.189	0.17	0.295	0.032*	0.432	0.001**
GCF total amount of IL-1β (pg/30 s)	0.576	< 0.001**	0.605	< 0.001**	0.521	< 0.001**	0.671	< 0.001**	0.574	< 0.001**	-0.294	0.03*	-0.180	0.19	0.272	0.049*	**-0.043**	**0.75**		
Salivary IL-10 (pg/ml)	0.105	0.45	0.155	0.26	0.103	0.46	0.118	0.40	0.114	0.41	0.074	0.59	0.208	0.13	-0.174	0.21				
GCF total amount of IL-10 (pg/30 s)	0.294	0.032*	0.138	0.32	0.187	0.18	0.235	0.09	0.134	0.34	-0.168	0.228	-0.356	0.009**						
Salivary Metrnl (pg/ml)	-0.160	0.25	-0.149	0.28	-0.326	0.01*	-0.283	0.04*	-0.366	0.007**	-0.029	0.83								
GCF total amount of Metrnl (pg/30 s)	-0.198	0.15	-0.234	0.09	-0.086	0.54	-0.213	0.12	-0.181	0.18										

r: Spearman’s rho correlation coefficient

* Correlation significance at the *p* < 0.05 level

** Correlation significance at the *p* < 0.01 level

GI, gingival index; PI, plaque index; BOP, bleeding on probing; PD, probing pocket depth; CAL, clinical attachment loss; GCF, gingival crevicular fluid; IL, interleukin

Salivary Metrnl concentrations were negatively correlated with %BOP, PD and CAL (*p* < 0.05). Salivary Metrnl concentrations was negatively correlated with GCF total amount of IL-10 (*p* = 0.009). GCF total amount of Metrnl were negatively correlated with GCF total amount of IL-1β concentrations (*p* = 0.03) (Table 3).

#### Receiver operating characteristic (ROC) curve analysis

**Fig. 1:**
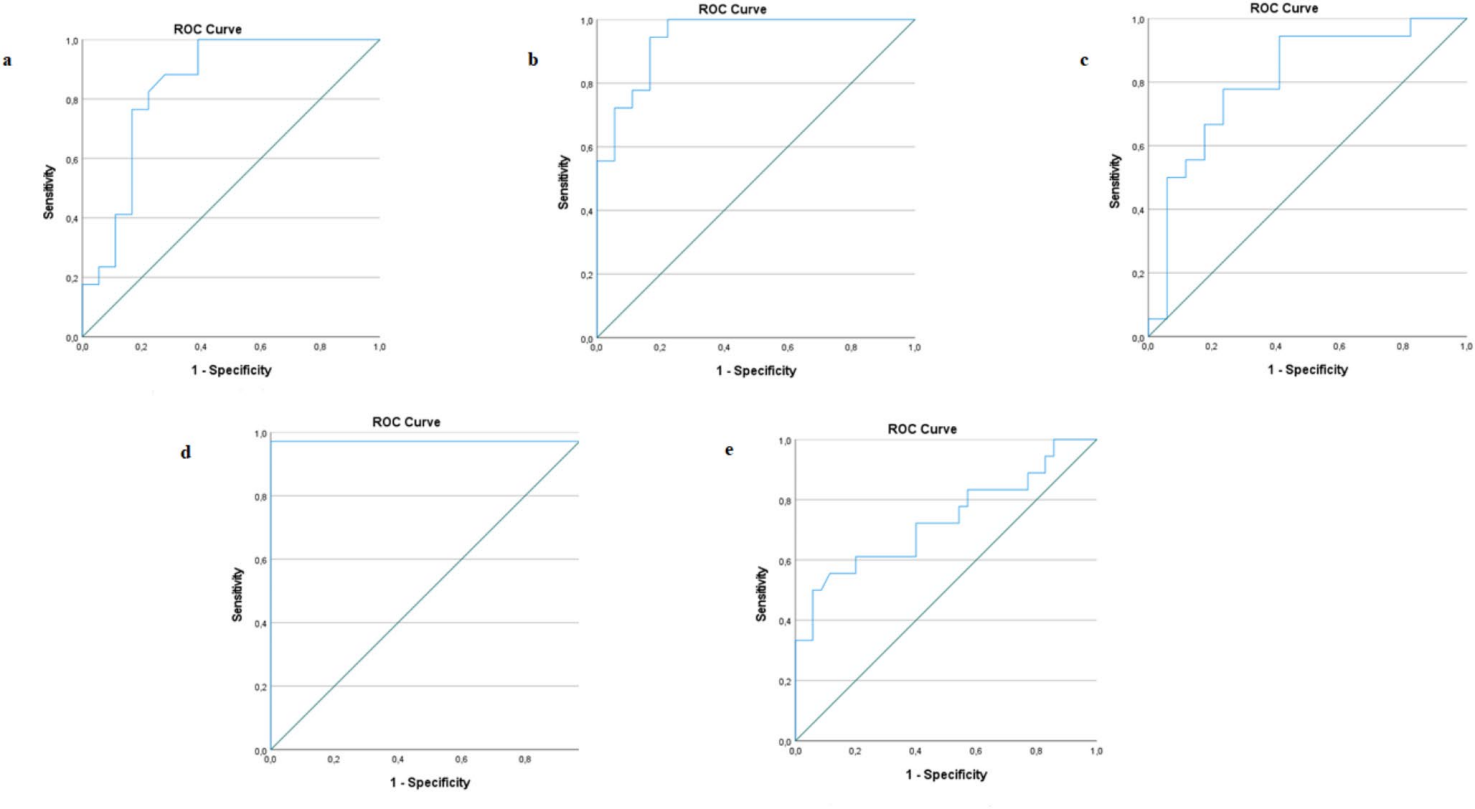
ROC curve analysis for: IL-1β and salivary Metrnl. **a** GCF total amount of IL-1β (pg/30 s); discriminate gingivitis patients from healthy subjects **b** GCF total amount of IL-1β (pg/30 s); discriminate periodontitis patients from healthy subjects **c** GCF total amount of IL-1β (pg/30 s); discriminate periodontitis patients from gingivitis patients **d** Salivary IL-1β (pg/mL); discriminate periodontitis and gingivitis patients from healthy subjects **e** Salivary Metrnl (pg/mL); discriminate healthy and gingivitis patients from periodontitis patients

ROC curve analysis are summarized in Table 4; Fig. 1. For each biomarker, groups with no significant difference were combined with each other. For salivary IL-1β concentrations, the difference between the control group and the combination of the other groups (gingivitis + periodontitis) was examined. For GCF total amount of IL-1β, pairwise comparisons were made between each group. For salivary Metrnl concentrations, the difference between the periodontitis group and the combination of the other groups (control + gingivitis) was examined (AUC = 0.731, cut-off value 98.59 (pg/mL), sensitivity = 72%, specificity = 60%, *p* < 0.006) (Table 4; Fig. 1).

**Table 4:** The data (ROC analysis) of IL-1β and metrnl sensitivity, and specificity for periodontal diseases

Test variable(s)	AUC (95% CI)	Cut-off value	*P*	Sensitivity (%)	Specificity (%)
Salivary IL-1β (pg/mL) Healthy vs. (Gingivitis + Periodontitis)	0.971 (0.916- 1.0)	21.05	< 0.001	97.1	100
GCF total amount of IL-1β (pg/30 s) Healthy vs. Gingivitis	0.845 (0.708–0.981)	4.48	< 0.001	100	61.1
GCF total amount of IL-1β (pg/30 s) Healthy vs. Periodontitis	0.944 (0.875- 1.0)	7.34	< 0.001	94.4	83.3
GCF total amount of IL-1β (pg/30 s) Gingivitis vs. Periodontitis	0.807 (0.657–0.957)	9.05	0.002	94.4	58.8
Salivary Metrnl (pg/mL) (Healthy + Gingivitis) vs. Periodontitis	0.731 (0.575–0.887)	98.59	0.006	72.0	60.0

Statistically significant at *p* < 0.05

SD, standard deviation; AUC, area under curve; GCF, gingival crevicular fluid; vs., versus

## Discussion

To the best of authors’ knowledge, this is the first study to assess Metrnl levels in periodontal disease. Salivary and GCF Metrnl, IL-1β, and IL-10 levels were evaluated in patients with stage III periodontitis. There was a significant decrease in salivary Metrnl concentrations of the periodontitis group and salivary Metrnl and IL-1β concentrations were significantly correlated with periodontal clinical parameters (negative and positive, respectively). The presence and decreasing levels of Metrnl in oral fluids suggested that these biomolecules are related with periodontitis.

Various cytokines or enzymes were identified as candidate biomarkers of the onset and progression of periodontal disease in saliva and GCF [47]. Non-invasively collected saliva is circulating fluid and provides information about periodontitis cases at the individual level [48]. GCF, however, reflects region-level changes as an exudate [49]. Thus, saliva and GCF samples were evaluated in our study. Consistently low GCF volumes in healthy regions can lead to overestimation of the concentrations of the molecules under study, resulting in misleading assessments [12]. Therefore, we presented and discussed GCF levels as total amount.

Stage I periodontitis represents the borderline between gingivitis and periodontitis, signifying the early stages of attachment loss, known as initial periodontitis. Stage II represents moderate periodontitis, while stages III and IV represent severe periodontitis [43]. A comparison of periodontitis patients in the 2018 versus 1999 classification revealed that the majority were at stage III [50]. Stage IV periodontitis is less common than stage III, and the increased number of tooth losses in stage IV poses challenges in terms of GCF supply [43, 51]. Based on this information, our study included individuals with generalized stage III periodontitis.

IL-1β is a well-known pro-inflammatory cytokine in periodontal diseases. The effects of IL-1β in the pathogenesis of periodontal disease are associated with increased tissue concentration [8]. Salivary IL-1β concentrations increase in periodontal disease [9–11, 52]. Similarly, in our study, salivary IL-1β concentrations were significantly higher in the periodontal disease groups. In some studies, although salivary IL-1β concentrations were elevated in the periodontitis group, there was no significant difference between the gingivitis and control groups [13] or between the periodontitis and the gingivitis groups [52, 53]. Similar to Ramseier et al. [53] and Afacan et al. [52], no significant difference was observed between periodontal disease groups in the present study.

Various studies have reported that the GCF total amount of IL-1β in the periodontitis group was statistically significantly higher than in the gingivitis and control groups, while no significant difference was detected between the gingivitis and control groups [12, 13]. Dikilitaş et al. [54] found no significant difference among the periodontal health, gingivitis, and Stage I periodontitis group in terms of GCF total amount of IL-1β. The GCF total amount of IL-1β in the Stage II, III, and IV groups, however, were found to be significantly higher than periodontal health, gingivitis, and Stage I periodontitis group. Yavuz et al. [10] found that total GCF total amount of IL-1β was significantly higher in the chronic periodontitis (Stages II, III, and IV) and gingivitis groups, but the values of the periodontitis group were significantly higher than the gingivitis group. Similarly, GCF total amount of IL-1β differed significantly among all groups in our study, and GCF total amount of IL-1β increased with deterioration of periodontal health.

In this study, salivary IL-1β concentrations were found to be higher in the gingivitis group than in the periodontitis group, although not significantly. However, the GCF total amount of IL-1β was significantly higher in the periodontitis group. Considering that GCF is one of the sources of IL-1β in salivary, individuals in the gingivitis group (mean missing teeth *n* = 0.18 ± 0.72) had fewer missing teeth than those in the periodontitis group (mean missing teeth *n* = 1.89 ± 1.74), resulting in more GCF containing IL-1β flowing into the salivary, potentially leading to increased salivary concentrations of IL-1β in the gingivitis group.

Our results were consistent with studies reporting increased salivary concentrations and GCF total amount of IL-1β in periodontal disease [9–11, 55–60]. Increased levels are associated with periodontal clinical parameters such as BOP, PD, and CAL. In accordance with the previous studies [10, 13, 59–63], salivary concentrations and GCF total amount of IL-1β had significant positive correlations with periodontal clinical parameters. In our study, the evaluation of IL-1β revealed its potential to distinguish between healthy and periodontal disease groups and reflect periodontal inflammatory status.

IL-10 can interfere with tissue homeostasis by pro-inflammatory cytokine suppression, thus playing a positive role in preventing the progression of periodontitis and increasing its stability [56]. Various studies have reported that salivary IL-10 concentrations in individuals with periodontitis increase [11], decrease [20], or do not differ [19, 53] compare to healthy controls. Similar to Rabelo et al. [16] in our study, although relatively higher salivary IL-10 concentrations were observed in the periodontitis group, the difference between the groups was not significant. Some studies report that GCF total amount of IL-10 increase [16, 17, 64], decrease [21] or no differ [18, 53] in periodontal disease compared to periodontally healthy controls. Bozkurt et al. [21] reported significantly lower GCF total amount of IL-10 in the periodontitis group. Although there was no significant difference among the groups in the present study, GCF total amount of IL-10 decreased in the gingivitis and periodontitis groups. Differences in the disease levels of the study populations, the IL-10 inhibitory mechanisms found in salivary and GCF, and differences in methodology such as sample collection, processing, and storage may have influenced this variation in the study results.

There are different results in studies evaluating plasma, synovial membrane, and serum Metrnl levels in systemic diseases or conditions associated with chronic inflammation. Decreased Metrnl plasma levels have been reported in chronic obstructive pulmonary disease [30], while increased levels have been found in type 2 diabetes [31]. This increase could be explained as a compensatory response [31]. In rheumatoid arthritis and in various skin diseases, such as atopic dermatitis and psoriasis, Metrnl expression was reported to be significantly up-regulated in synovial membranes [26]. Additionally, increased [32, 33] or decreased [34–36] serum Metrnl expression levels were reported in various systemic diseases such as inflammatory bowel disease, obstructive sleep apnea syndrome, rheumatoid arthritis, coronary artery disease, type 2 diabetes, and in the early phase of sepsis. We are unaware of any study evaluating Metrnl levels in the presence of periodontal inflammation.

Metrnl plays a role in immune cells as well as in inflammation [22]. In a sepsis model, Treg cells, which play a role in suppressing inflammation by preventing excessive reaction, were reduced in peripheral blood samples of Metrnl^−/−^ mice. Impaired immune defense in sepsis was associated with decreased macrophage recruitment and Treg/Th17 imbalance [33]. In another study, Metrnl^−/−^ mice were found to be prone to developing inflammatory lesions, which was interpreted as Metrnl deficiency possibly supporting the development of inflammation [22]. Overexpression of Metrnl plays an anti-inflammatory role through AMPK-PAK2 signaling and inhibits inflammation in injured H9C2 cells. Jung et al. [28] demonstrated that Metrnl treatment could increase AMPK phosphorylation and PPARδ expression, thereby reducing the inflammatory response through NF-κB-mediated signaling and suppression of pro-inflammatory cytokines. In an experimental allergic asthma study examining the role of Metrnl in alleviating DC-mediated type 2 inflammation, Metrnl was a regulator with anti-inflammatory activities [65]. Only one study evaluating salivary Metrnl concentrations has been found. No difference was observed between the salivary Metrnl concentrations of the control group and of patients with newly-diagnosed type 2 diabetes. After three months of metformin treatment, salivary Metrnl concentrations increased significantly in the diabetic group [29]. Although there was no significant difference between the gingivitis and healthy groups in our study, salivary Metrnl concentrations were higher in the gingivitis group. However, salivary Metrnl concentrations were significantly lower in the periodontitis group. This suggests that Metrnl may have increased in the early phase of periodontal disease by exerting anti-inflammatory properties to balance the inflammatory phase as a result of the possible immunoregulatory effect of Metrnl, while in periodontitis, which is the chronic phase, Metrnl may have been decreased in the long term by undergoing cleavage due to increased protease. This decrease in the anti-inflammatory response supports the idea that susceptibility to periodontitis may be related to the increase in pro-inflammatory mediators as well as the decrease in anti-inflammatory pathways [66].

In our study, although GCF total amount of Metrnl was relatively lower in the gingivitis and periodontitis groups, no significant difference was found among the groups. Metrnl is released intensely from the mucosa, one of the barrier membranes [26]. In addition, Metrnl immunoreactivity was shown in the intra- and interlobular channels of the parotid and submandibular glands [29]. In our study, a significant decrease was observed in salivary Metrnl concentrations in the periodontitis group, which was not evident in GCF total amount of Metrnl. This may be due because the interaction of salivary content with the oral mucosa is greater than that of GCF, and that saliva is a fluid that includes parotid and submandibular gland contents as well as GCF.

Metrnl increases the expression of anti-inflammatory genes such as IL-10 and transforming growth factor-β, while causing a decrease in the expression of pro-inflammatory genes such as tumor necrosis factor‑α (TNF-α), Interferon-γ, and IL-1β [67]. However, in individuals with inflammatory bowel disease [35], type 2 diabetes, and coronary artery disease [36] serum Metrnl levels showed a significant negative correlation with serum inflammatory cytokines (TNF-α, IL-6). Chen et al. [33] reported that serum Metrnl levels had a negative correlation with IL-1β in septic patients in an intensive care unit. GCF total amount of Metrnl and GCF total amount of IL-1β levels also showed a negative correlation in our study. Additionally, significant negative correlations were observed between salivary Metrnl concentrations and periodontal clinical parameters (PD, %BOP, and CAL). In ROC analysis, salivary Metrnl showed significant predictive value in diagnosing periodontitis. Based on these findings, it can be concluded that salivary Metrnl concentrations may be related to the presence and severity of periodontal disease.

As no study has yet evaluated Metrnl concentrations in the presence of periodontal inflammation, we were not able to directly compare Metrnl concentrations, but our findings may serve as a reference point for future studies. The major limitation of our study include the inability to establish a cause-effect relationship due to the cross-sectional study design. Another limitation, include the relatively limited number of participants. Finally, the changes in the concentrations of these inflammatory mediators after periodontal treatment could not be investigated, also due to the study design.

## Conclusion

Within the limitations of this study, a significant decrease in salivary Metrnl concentrations was found in patients with periodontitis compared to the healthy group. ROC analysis indicates that salivary Metrnl concentrations may provide significant predictive value for periodontitis. Our findings suggest that salivary Metrnl concentrations may serve as a potential biomarker of periodontitis. Further randomized controlled clinical studies with larger sample sizes are necessary to fully elucidate the functional impact of Metrnl on periodontal inflammation.

## Data Availability

No datasets were generated or analysed during the current study.
